# ADAP1/Centaurin-α1 Negatively Regulates Dendritic Spine Function and Memory Formation in the Hippocampus

**DOI:** 10.1523/ENEURO.0111-20.2020

**Published:** 2021-01-05

**Authors:** Erzsebet M. Szatmari, Corey Moran, Sarah Cohen, Amanda Jacob, Paula Parra-Bueno, Naomi Kamasawa, Debbie Guerrero-Given, Michael Klein, Robert Stackman, Ryohei Yasuda

**Affiliations:** 1 Max Planck Florida Institute for Neuroscience, Jupiter, FL 33458; 2 College of Allied Health Sciences, East Carolina University, Greenville, NC 27834; 3 Florida Atlantic University, Jupiter, FL 33458

**Keywords:** ADAP1/Centaurin-α1, Arf6, dendritic spines, hippocampus, learning and memory

## Abstract

ADAP1/Centaurin-α1 (CentA1) functions as an Arf6 GTPase-activating protein highly enriched in the brain. Previous studies demonstrated the involvement of CentA1 in brain function as a regulator of dendritic differentiation and a potential mediator of Alzheimer’s disease (AD) pathogenesis. To better understand the neurobiological functions of CentA1 signaling in the brain, we developed *Centa1* knock-out (KO) mice. The KO animals showed neither brain development nor synaptic ultrastructure deficits in the hippocampus. However, they exhibited significantly higher density and enhanced structural plasticity of dendritic spines in the CA1 region of the hippocampus compared with non-transgenic (NTG) littermates. Moreover, the deletion of *Centa1* improved performance in the object-in-place (OIP) spatial memory task. These results suggest that CentA1 functions as a negative regulator of spine density and plasticity, and of hippocampus-dependent memory formation. Thus, CentA1 and its downstream signaling may serve as a potential therapeutic target to prevent memory decline associated with aging and brain disorders.

## Significance Statement

ADAP1/Centaurin-α1 (CentA1) is highly enriched in the brain and has been shown to be involved in the developmental regulation of dendritic differentiation. Although increased CentA1 level has been linked to Alzheimer’s disease (AD), the underlying neurobiological mechanisms are unknown. We found that the genetic deletion of *Centa1* leads to increased dendritic spine density and enhanced spine structural plasticity in the hippocampus, accompanied by behavioral improvements in a location recognition task. This is the first study of CentA1 role in the brain, and our findings will facilitate the understanding of neurobiological mechanisms underlying the regulation of dendritic spine morphology and plasticity in healthy brains and in neurobiological disorders.

## Introduction

Cumulated evidence indicates that structural plasticity of dendritic spines is essential for learning and memory (Colgan and Yasuda, 2014; Yasuda, 2017). Abnormal spine morphology and density are often associated with brain disorders, including Alzheimer’s disease (AD), suggesting the importance of spine plasticity in normal brain function (Jacobsen et al., 2006; Smith et al., 2009; van Spronsen and Hoogenraad, 2010; Wei et al., 2010; Perez-Cruz et al., 2011; De Strooper and Karran, 2016; Boros et al., 2019). Previously we identified that ADAP1/Centaurin-α1 (CentA1) is upregulated by amyloid β (Aβ), and this upregulation correlates with Aβ-dependent neuronal dysfunction, including spine loss and deficits in spine structural plasticity in rodents (Szatmari et al., 2013). Furthermore, increased intracellular level of CentA1 and enhanced association with neuritic plaques has been reported in postmortem human AD brain (Stricker and Reiser, 2014). Thus, CentA1 may be involved in AD progression.

CentA1 is almost exclusively expressed in the brain, with the highest level in the amygdala, hippocampus, and hypothalamus (Sedehizade et al., 2002). Its expression in the brain is developmentally regulated, reaching its peak at postnatal weeks 2–4 in mice, followed by a reduction in adulthood (Moore et al., 2007). Although earlier reports indicated that CentA1 localization in the brain is restricted to neurons (Stricker and Reiser, 2014), a recent study suggested that glial cells also express this protein (Zhang et al., 2016). At the subcellular level, CentA1 is present in axonal processes (Kreutz et al., 1997; Stricker et al., 1997), in dendrites and dendritic spines, in the postsynaptic density (PSD; Yoshimura et al., 2004; Moore et al., 2007), at the plasma membrane, in the nucleus, and in mitochondria (Stricker and Reiser, 2014).

CentA1 is a multidomain protein with an ArfGAP domain and two PH domains. The ArfGAP domain can interact *in vitro* with Arf1, Arf5, and Arf6, but *in vivo* it is selective toward Arf6 (Thacker et al., 2004; Venkateswarlu et al., 2004). In addition, multiple studies indicated that CentA1 functions as a Ras-anchoring protein involved in the activation of the ERK1/2 signaling pathway (Hayashi et al., 2006; Szatmari et al., 2013). Consistent with its important function in small GTPase signaling and high expression during development, it has been suggested that CentA1 is important for neuronal differentiation and development (Moore et al., 2007).

Previously, we reported that treatment of dissociated neurons or hippocampal slice cultures with Aβ induced transient upregulation of CentA1, followed by a reduction in dendritic spine density and abolishment of spine structural plasticity in organotypic hippocampal slices (Szatmari et al., 2013). Furthermore, when CentA1 is downregulated with shRNA, these cellular phenotypes were significantly suppressed. These findings suggest that CentA1 contributes to Aβ-dependent neuronal dysfunction associated with AD. However, the roles of CentA1 in synaptic plasticity *in vivo* are unknown.

To determine the function of CentA1 in the brain, we created mice lacking CentA1. In the present study, we provide evidence that the deletion of CentA1 in the brain leads to an increased density of dendritic spines in the hippocampus and to the enhancement of dendritic spine structural plasticity induced by two-photon glutamate uncaging. We also show that the lack of CentA1 does not affect the ultrastructure of synapses in the hippocampus. Furthermore, these animals showed increased performance in the object-in-place (OIP) spatial memory test. Taken together, our results indicate that CentA1 is a negative regulator of dendritic spine density and plasticity and, consequently, of learning and memory function.

## Materials and Methods

### Mice

All mice were housed in the Animal Resource Facility of Max Planck Florida Institute for Neuroscience, compliant with the National Institutes of Health *Guide for Care and Use of Laboratory Animals*.

**Figure 1.**
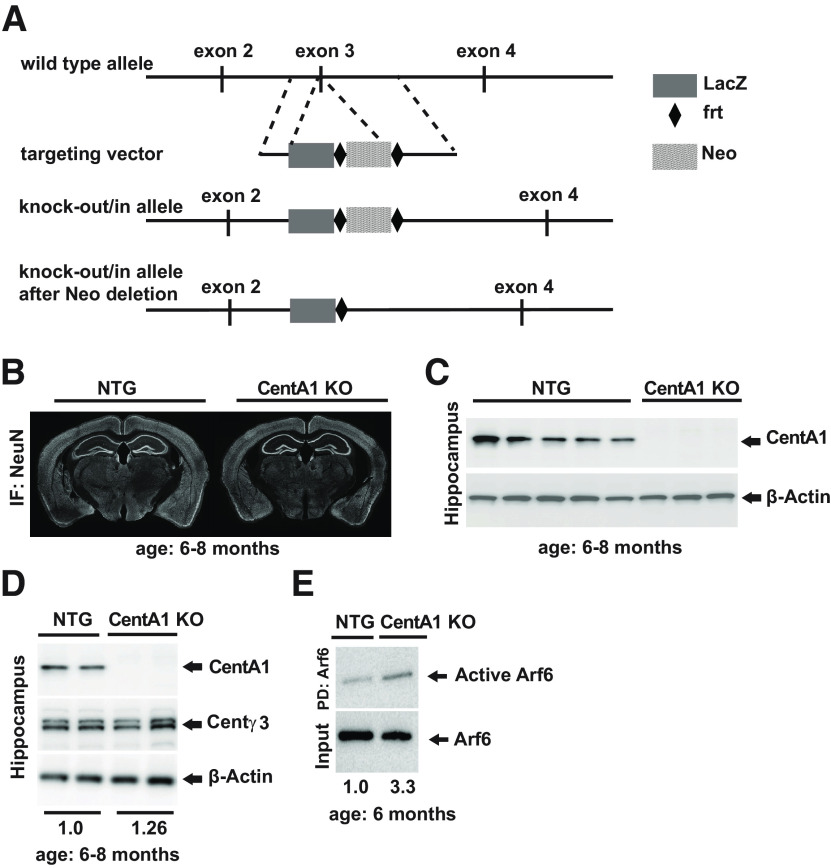
Generation of CentA1 KO mutant mice. ***A***, Schematic drawing of the targeting strategy used to generate CentA1 global KO mouse lines. Exon 3 of *Centa1* gene was replaced by sequences for LacZ, followed by a translational STOP, and a Neo selection cassette, flanked by *frt* sites. Expression and translation of this modified *Centa1* locus resulted in a fusion protein of the beginning of CentA1 and LacZ, while functional CentA1 protein is lacking. ***B***, NeuN immunohistochemistry of coronal sections from six- to eight-month-old CentA1 KO and NTG littermate mice show normal brain morphology in CentA1 KO mice. ***C***, Immunoblots show the complete lack of CentA1 protein in the hippocampus from CentA1 KO mice. Bottom, Anti-β-Actin antibody shows that a similar amount of protein samples were loaded between genotypes. NTG: *n* = 5 mice; CentA1 KO: *n* = 3 mice. ***D***, Immunoblots show that in the hippocampus of CentA1 KO mice, the level of another brain enriched Centaurin (Centγ3) does not undergo significant compensatory upregulation. The numbers under blots show hippocampal Centγ3 level normalized to β-Actin. NTG: *n* = 4 mice; CentA1 KO: *n* = 4 mice. Two-tailed *t* test, *p* = 0.29. ***E***, Immunoblots show the level of Arf6 activation in the hippocampi of six-month-old CentA1 KO and NTG littermate mice, evaluated by active Arf6 pull-down assay. Bottom, Anti-Arf6 antibody shows a similar amount of total Arf6 protein in the hippocampal lysates between genotypes. Numbers under blots represent the level of active (GTP bound) Arf6 normalized to total Arf6 protein in the hippocampal tissue. NTG: *n* = 2 mice; CentA1 KO: *n* = 3 mice.

CentA1 knock-out (KO) mice were generated from C567BL/6 mice at Duke University Transgenic Core Facility. A targeting vector for BAC recombineering was generated with 300-bp homology regions located upstream and downstream of exon three of the Centa1 locus. The homology regions in the vector were flanking sequences encoding for lacZ (PCR amplicon, using plasmid pRTHSP70-lacZ as a template), followed by the bGH polyadenylation sequence, and an frt site-flanked neomycin cassette (Fig. 1*A*). The sequences were homologously inserted into a *Centa1* locus-containing BAC (RP23-327I20; CHORI) through homologous recombination in *Escherichia coli* (recombineering; Yu et al., 2000), thereby replacing exon 3. Subsequently, an embryonic stem cell targeting vector containing the modified *Centa1* locus was retrieved through recombineering from the BAC into a plasmid vector (PL253; Liu et al., 2003). This was followed by homologous targeting of the *Centa1* locus in 129 Sv ES cells (R1; Nagy et al., 1993) by electroporation of the linearized targeting vector, selection of neo-resistant colonies, and PCR screening for homologous integration of the targeting vector by detection of an amplicon using primers within the lacZ sequence and outside the short homology arm. Correctly modified ES cells were then injected into C57BL/6 blastocysts using standard procedures. Chimeric males were mated with black wild-type females (C57BL/6) to generate CentA1 heterozygote mice.

All experiments were conducted on male mice, according to the institutional ethical guidelines for animal experiments. The genotype of each mouse used for experiments was verified by PCR of genomic DNA isolated from tail DNA before experiments and by Western blotting of brain samples after experiments.

In all experiments, controls are non-transgenic (NTG) littermates of the CentA1 KO mice, to ensure that the mice are from the same parents, in the same cage and are uniformly affected by any environmental effects. In experiments involving adult animals, we used only male mice to prevent hormone-induced data variability in females because of the estrous cycle.

### Organotypic hippocampal slice culture preparation and treatment

Organotypic hippocampal slice cultures were prepared at postnatal day 5 or 6 from CentA1 KO mice and their NTG littermates in accordance with the animal care and use guidelines of Max Planck Florida Institute for Neuroscience. Pups of both sexes were used for slice culture preparation. Briefly, the mouse was deeply anesthetized with isoflurane, followed by quick decapitation and isolation of the brain. The hippocampi were dissected in dissection medium (for 1 l: 1 ml of 1 m CaCl_2,_ 5 ml of 1 m MgCl_2,_ 1.8 g of D-glucose, 4 ml of 1 m KCl_,_ 1.7 g NaHCO_3_, 84.8 g sucrose, and 5.98 g HEPES) and sliced into 350-μm-thick sections using a McIlwain tissue chopper. Hippocampal slices were cultured on tissue culture inserts (Millicell) placed in six-well tissue culture plates with tissue culture medium (for 2.5 l: 20.95 g MEM, 17.9 g HEPES, 1.1 g NaHCO_3,_ 5.8 g D-glucose, 120 μl of 25% ascorbic acid, 30 ml Glutamax, 2.5 ml insulin, 500 ml heat-inactivated horse serum, 5 ml of 1 m MgSO_4_, and 2.5 ml of 1 m CaCl_2_). The tissue incubator was set to 37°C and 5% CO_2_. After one week in culture, slices were biolistically transfected with eGFP. For ballistic gene transfer, gold particles (12 mg) were coated with plasmid DNA (50 μg total) and shot into slices using the Helios gene gun system (Bio-Rad).

### Image acquisition and two-photon glutamate uncaging

A custom-built two-photon microscope with two Ti: sapphire lasers (Spectra-physics) was used. One laser was tuned at a wavelength of 920 nm to excite eGFP. All samples were imaged using <2 mW laser power measured at the objective (60×, 0.9 numerical aperture; Olympus). The second laser was tuned to 720 nm to uncage 4-methoxy-7-nitroindolinyl-caged-l-glutamate (MNI-caged glutamate) with a train of 6 ms, 5 mW pulses (30 times at 0.5 Hz) near the head of the spine of interest. The beams were combined and passed through the same set of scan mirrors and objective. Fluorescence signal from a cooled PMT (Hamamatsu) was acquired using a data acquisition board controlled by Scanimage software. Two-photon glutamate uncaging was performed at 24–26°C (room temperature) in Mg^2+^-free artificial CSF (ACSF; 127 mm NaCl, 2.5 mm KCl, 4 mm CaCl_2_, 25 mm NaHCO_3_, 1.25 mm NaH_2_PO_4_, and 25 mm glucose) containing 1 μm tetrodotoxin (TTX) and 4 mm MNI-caged glutamate aerated with 95% O_2_ and 5% CO_2._ We imaged one to five spines/neuron and at least two neurons/animal.

### Analysis of spine density and structural plasticity

Spine density analyzes were performed on secondary apical dendrites of CA1 pyramidal neurons in organotypic hippocampal slices transfected with eGFP and imaged live with two-photon microscope. The number of spines/100 μm was determined using ImageJ software. Spine volume was reported using the green fluorescent intensity from eGFP and was measured as integrated fluorescent intensity after background subtraction (*F*). Spine volume change was calculated by *F*/*F*_0_, in which *F*_0_ is the average spine volume measured over 15 min before stimulation. Spine density was evaluated in parallel with sLTP on slices from the same mice, using Fiji imaging analysis software (http://fiji.sc/Fiji). The person performing spine density and spine volume change analysis was blinded to genotype.

### Arf6 activation assay

Basal Arf6 activity level in CentA1 KO mice and their NTG littermates was determined using Cell Biolabs’ Arf6 activation assay kit and following the manufacturer’s instructions. Briefly, the active form of Arf6 from hippocampal lysates was selectively isolated and pulled down with the GGA3 protein-binding domain (GGA3-PBD) attached to agarose beads. The precipitated GTP-bound, and therefore active Arf6, was detected by Western blotting using an anti-Arf6 antibody.

### SDS-PAGE and immunoblotting

Hippocampi were extracted with T-PER protein extraction buffer (Pierce) supplemented with inhibitors for proteases and phosphatases (Roche). The lysates were centrifuged at 15,000 × *g* for 15 min at 4°C, and the supernatants were used for further analysis. Samples were prepared for standard SDS-PAGE and separated on 4–20% gradient acrylamide gel (Mini-PROTEAN TGX precast gels, Bio-Rad), then transferred onto 0.45 μm pore size PVDF membranes (Millipore) using semi-dry immunoblotting (transfer buffer containing 25 mm Tris, 200 mm glycine, and 20% methanol). Membranes were blocked with 5% nonfat milk (Great Value) in Tris-buffered saline with 0.1% Tween 20 (TBS-T) for 1 h at room temperature, then incubated overnight at 4°C with primary antibodies diluted in 5% BSA in TBS-T. We used the following commercially available antibodies: goat anti-Centaurin-ɑ1 (Abcam; 1:500), rabbit anti-Centaurin γ3 antibody (Santa Cruz Biotechnology; 1:500), mouse anti-β-Actin (Sigma, 1:1000); rabbit anti-Arf6 antibody (Cell Signaling Technologies; 1:1000), and mouse anti-Ras antibody (Thermo Fisher Scientific; 1:1000). Membranes were washed three times for 15 min in TBS-T, followed by incubation for 2 h at room temperature with horseradish peroxidase-conjugated goat anti-rabbit or rabbit anti-mouse secondary antibodies (Bio-Rad), diluted 1:5000 in 5% nonfat milk in TBS-T. Membranes were washed three times for 15 min in TBS-T, then incubated with Pierce ECL Plus Western blotting substrate or Pierce ECL Western blotting substrate (for β-Actin) to detect Western blotted proteins. We used the Image Quant LAS4000 Imaging System (GE Healthcare) to visualize protein bands. ImageJ software was used for Western blot quantification.

### Behavioral studies

Behavioral assays were performed at the Scripps Florida Institute Behavioral Core Facility, and at Florida Atlantic University. We performed a battery of behavioral tests that included open field (OF), OIP, elevated plus maze (EPM), spontaneous alternation (SA), Rota-rod (RR), and Morris water maze (MWM). All mice tested were included in data analyses, performed by a person blinded to genotype.

#### OF test

In order to test for baseline activity, locomotor behavior was measured in 17 × 17-inch square acrylic open-field chambers. Before testing, uniformity of light across the arena was confirmed using a light intensity meter, and the chambers were cleaned with 1% Micro-90 before and between trials. Background white noise (∼72 dB) was used during trials. Mice were placed into the center of the chamber to begin testing, and activity was recorded for 30 min. Data were analyzed in 10-min blocks.

#### EPM test

An EPM test was used to assess baseline anxiety-like behavior. Mice were placed in the center of the plus maze (Med Associates) and could explore the maze for 5 min. Time spent in open and closed arms, number of arm entries, latency to initially enter an open arm, and total distance moved were recorded using EthoVision XT (Noldus Information Technology Inc.). Uniformity in lighting was confirmed across the maze, and the maze was cleaned with 1% Micro-90 before each trial. Background white noise (∼70 dB) was used during trials.

#### SA test

Working memory was assessed in a SA test. Mice received two tests, each separated by 3 days. Two mazes were used, each turned in a different configuration, to increase novelty to the maze on the second test. Each maze contained three arms with walls made opaque, including a start box (17.8 × 7.3 cm) at the base of the start arm (38.1 × 7.3 cm) and adjoined to a central choice area (10.2 × 10.2 cm) with two choice arms (30.5 × 7.3 cm) radiating 180 degrees from the central choice area (forming a “T”). Automatic guillotine doors were installed at the entry of each arm that was controlled by EthoVision XT (Noldus Information Technology Inc.). Each test was conducted as follows: a mouse was placed in a start box, and the door to the maze subsequently opened, allowing the mouse to enter the maze and explore to the T intersection. Upon reaching the intersection, the mouse chose an arm (free choice trial) and, after three body points had entered that arm, the door closed automatically, detaining the mouse in that arm for a period of 10 s. During those 10 s, a cloth lightly sprayed with 70% ethanol was used to wipe the maze outside the chosen arm to remove possible odor cues. After 10 s, the mouse was placed back in the start box for a second free choice trial, after which the door to that arm again closed, detaining the mouse in that arm until prompt removal. Of the two tests the mouse was given, one allowed the mouse to immediately enter the maze for the second trial (no delay trial), and one kept the mouse in the start box for 60 s before the door opened to allow the mouse to enter the maze for a second trial (delay trial). Groups were balanced for maze, test day, and delay. Alternation success was calculated for each test. If a mouse did not leave the start box to enter the maze after 60 s, it was gently nudged with a cotton swab. The maze was cleaned with 70% ethanol between mice. Background white noise (∼70 dB) was used during trials.

#### RR test

The RR test was used to measure motor coordination and balance. Briefly, five mice were tested simultaneously on an accelerating RR (Med-Associates) set to accelerate from 4 to 40 rpm over 5 min. Mice were tested for three trials with an intertrial interval of no less than 30 min. A beam break occurred when a mouse fell from the rotating rod, signaling the timer to stop automatically. The time when an animal fell from the rod was recorded. A mouse was removed from the rotating rod if it clung to the rod and completed more than two “full passive rotations” or if it completed up to two full passive rotations in more than two separate bouts. The time at removal was recorded. Trials were averaged for data analysis. The RR was cleaned with 70% ethanol between trials, and background white noise (∼70 dB) was used during trials.

#### MWM test

The MWM was performed to assess spatial learning and memory. Briefly, the water maze consisted of a 1.4-m diameter white tank with a 10-cm diameter platform submerged ∼1 cm below the surface of the water. The water was made opaque using non-toxic white washable paint that made the platform invisible during trials. The temperature of the water was kept at 22–24°C. Visual cues were placed in the testing room around the tank for spatial reference. Before water maze training, mice received a visual platform test where the spatial cues were removed, and the platform was elevated above the surface of the water and marked with a cue, so it could clearly be discerned. Mice were given four trials, and the platform location was varied over trials. This served to verify the visual ability of the mice and to ensure that the mice had no deficits that would affect their ability to swim to the platform. For the hidden platform test, mice were given four acquisition trials per day for eight consecutive days. The start location was varied for each trial, and the mice were allowed 60 s to find the platform. Mice were left on the platform for 15 s before removing them from the water maze. If a mouse did not find the platform within 60 s, it was placed on or guided to the platform and kept there for 15 s. Mice were dried after each trial and placed into cages located atop heating pads to prevent hypothermia. Daily acquisition trials were averaged for analysis. On day 9, mice were given a probe test during which the platform was not present. Activity and performance were tracked using EthoVision XT (Noldus Information Technology). Total time spent in each quadrant, the total number of entries into the target quadrant, the total number of platform crossings, latency to first platform crossing, and average distance to the platform center were recorded.

#### OIP memory test

The apparatus consisted of two open-top, high-walled square arenas made of white ABS (each: 37.5 × 37.5 × 50.0 cm). A salient landmark cue (blue plastic tarp, 20.3 × 25.4 cm) was affixed with clear tape to the center of the north wall. Each mouse was habituated to one of the arenas for 10 min/day for two consecutive days. On days 3 and 4, each mouse was returned to the familiar arena that now contained two novel toy objects (stainless steel cabinet leveling foot attached to a Plexiglas base, 4.2-cm diameter and 6.0 cm tall; metal spring attached to a Plexiglas base, 2.0-cm diameter and 4.8 cm tall) for 10-min training sessions. The two objects were positioned on the arena floor 2 cm from the corners on either side of the landmark cue (NW and NE). During the test session 24 h later (day 5), each mouse was given a 5-min test session in the familiar arena, yet one of the toy objects was transferred to the opposing southern corner (Fig 5*C*). The objects and the arena floor and walls were cleaned with 70% ethanol after each session. All behavioral testing data were digitally acquired by the EthoVision XT (Noldus Inc.) software package. Object exploration was scored off-line from the digital video files by experimenters that were blind to the genotype of the mice. OIP memory was inferred from the discrimination ratio, calculated for each subject by subtracting the time spent exploring the familiar object in the familiar location from the time spent exploring the familiar object in the novel location and then dividing the result by the total time spent exploring both objects. Discrimination ratios range from −1 to 1, with 0 indicating chance performance, a lack of preference for one object location over another, and positive ratios indicating novel object location preference. During training, mice that did not explore the objects for a minimum of 50 s were excluded from analyses. The data for mice that did not explore the objects for a minimum of 20 s during the test session were also excluded from all analyses.

### Golgi–Cox staining

We used a commercially available Golgi–Cox staining system, the FD Rapid Golgi Staining kit, which provides an adequate number of well-impregnated neurons with clearly visible spines. Briefly, mice were deeply anesthetized with ketamine/xylazine cocktail until lack of response to toe pinch was recorded. The brain was quickly collected from the skull and rinsed with Milli-Q water to remove blood from the surface. Impregnation was performed following the manufacturer’s instructions. Further processing of samples was done at FD Neurotechnologies. Images of pyramidal neurons from the CA1 area of the hippocampus were collected on 100 μm-thick slices using a Zeiss 780 confocal laser-scanning microscope. In order to detect Golgi–Cox staining, the microscope was set up in transmission mode, and 488 nm wavelength laser was used. Confocal images were obtained using a Plan-Neofluar 63× water (1.3 numerical aperture) objective. Each frame was acquired eight times and then averaged to obtain noise-free images. Spine density was measured by a person blinded to genotype and using Fiji imaging analysis software (http://fiji.sc/Fiji).

### Immunofluorescence staining

Adult male mice (four to seven months old) were deeply anesthetized with ketamine/xylazine cocktail until lack of response to toe pinch was recorded, then perfused transcardially with saline followed by perfusion with 4% paraformaldehyde (PFA) in 0.1 m phosphate buffer (PB). Brains were removed and post fixed overnight in the same fixative at 4°C. Coronal sections were cut at 50 μm on a Leica vibratome and collected in ice-cold 0.1 m PB. After a brief rinse with 0.1 m PB, the free-floating sections were incubated for 30 min in blocking buffer (0.3% Triton X-100 and 0.5% normal goat serum in 0.1 m PB). Sections were reacted overnight with anti-NeuN antibody (ABN78, rabbit polyclonal, Millipore) diluted 1:1000 in blocking buffer. Sections were washed in 0.1 m PB and then incubated for 2 h with Alexa Fluor 488-conjugated secondary antibodies (A-11008, Life Technologies) diluted 1:500 in blocking buffer. Sections were rinsed in 0.1 m PB, and the nuclei were stained with Hoechst (1:10,000, H3570, Life Technologies) for 10 min. Sections were rinsed again in 0.1 m PB and mounted on Superfrost plus slides (Thermo Fisher Scientific) using Fluoromount-G. We imaged four sections from four mice for both genotypes using a Zeiss LSM 710 confocal microscope.

### Electron microscopy (EM) studies

Male mice at seven to eight months of age were deeply anesthetized using euthasol (pentobarbital; 150 mg/kg, i.p.). Following lack of response to toe pinch, mice were perfused transcardially with warm PBS (150 mm NaCl, 25 mm Sorensen’s PB; pH 7.4), then with warm fixative solution (4% PFA, 2.5% glutaraldehyde in 100 mm Sorensen’s PB pH 7.4) for 7–9 min. Brains were postfixed with 4% PFA in PB for 2–4 h. After overnight washing with PB, 100 μm thick coronal sections were cut on a Leica VT1200 vibratome. Sections were briefly washed with water, treated with 1% aqueous OsO_4_ containing 1.5% potassium ferrocyanide for 40 min at 4°C, washed with water, followed by 1% aqueous OsO_4_ for 1 h at 4°C. Slices were then en bloc stained with 1% uranyl acetate for 25 min at 4°C, dehydrated in an increasing series of ethanol, acetone, and propylene oxide, and flat embedded in Durcupan resin (Sigma-Aldrich); 70 nm thick sections were prepared using an EM UC7 Leica ultramicrotome, followed by counterstaining with 3% uranyl acetate and 0.5% lead citrate. Sections were examined in a Tecnai G2 Spirit BioTwin transmission EM (TEM; Thermo Fisher Scientific) at 100-kV accelerating voltage. A Veleta CCD camera (Olympus) operated by TIA software (Thermo Fisher Scientific) was used for image acquisition at a magnification of 20,500× and 60,000×, respectively.

### TEM image acquisition and data analysis

All TEM data were analyzed using Fiji imaging analysis software (http://fiji.sc/Fiji). For unbiased synaptic spine analysis, we used the physical dissector method as described earlier (Sterio, 1984; Aziz et al., 2014). Briefly, we analyzed synaptic ultrastructure in the stratum radiatum (SR) of the hippocampus CA1 region, on spines of secondary apical dendrites located at 30–50 μm in the distance from the soma. A total of 20 images were acquired at 20,500× magnification, covering a total area of 478.2 μm^2^ per animal (20 ROIs). Each ROI was imaged from two consecutive sections that were used as “lookup” and “reference” section, respectively. Only synapses present in the lookup section but not in the reference section were counted. The following mathematical formula was used for estimation of spine density: spine density = *N*/*t*A (where *N* = the total number of spines counted in the lookup section; *t* = section thickness; A = area of the counting frame). On single EM images acquired at 60,000× magnification, we measured the length of PSD using the line tool and the area by outlining the electron density of the PSD using the polygon tool; the size of dendritic spine head; the length of the active zone (AZ); and the number of docked synaptic vesicles. AZ was defined as the presynaptic membrane directly opposing the PSD. SVs within 200 nm from the AZ were manually selected, and their distance from AZ was calculated using a 32-bit Euclidean distance map generated from the AZ (Montesinos et al., 2015). Vesicles located within 5 nm or less from the AZ membrane were considered “docked” (Yang et al., 2010). A person blinded to genotype analyzed three animals for each genotype. For each genotype, we analyzed 50 individual spine synapses per animal.

### Functional long-term potentiation (LTP)

We used CentA1 KO mice and their NTG littermates at six months of age for these experiments. Animals were sedated by isoflurane inhalation, then perfused intracardially with chilled choline chloride solution (124 mm choline chloride, 2.5 mm KCl, 26 mm NaHCO_3_, 3.3 mm MgCl_2_, 1.2 mm NaH_2_PO_4_, 10 mm glucose, and 0.5 mm CaCl_2_, pH 7.4 equilibrated with 95%O_2_/5%CO_2_). The brain was isolated and placed in choline chloride solution. Coronal slices were cut at 400 μm and maintained in oxygenated ACSF in a submerged chamber at 32°C for 1 h and then at room temperature during recording. Extracellular field potentials were recorded using 5- to 8-mΩ glass micropipettes filled with ACSF. Concentric bipolar stimulation electrode and the glass micropipette were placed into the SR of the CA1 area at ∼300 μm apart and at the same distance from the pyramidal cell layer and at the same depth. Signals were amplified at gain 100, filtered at 2 kHz and digitized at 20 kHz. An input/output curve was recorded to determine the stimulation amplitude, defined by the stimulus intensity that elicits a field EPSP (fEPSP) that is 50% of the supramaximal response. Stimulation was applied every 20 s, and the baseline was recorded for 20 min or until stabilization of the fEPSP amplitude. LTP was induced by high-frequency stimulation protocol consisting of three trains of 100 Hz with a 20 s interval between them. Poststimulus fEPSPs were then recorded for 1 h. Data analyzes was performed using an in-house program written with MATLAB.

### Experimental design and statistical analysis

For all experiments, CentA1 KO and their NTG littermates were processed in parallel. Specific sexes (males only) were used for adult behavior studies, electrophysiological recordings, Golgi staining and ultrastructural studies. Mix sexes were randomly used for all experiments involving organotypic hippocampal slice cultures. Excel, MATLAB, “R” software, and Prism 8 (GraphPad Software) were used for statistical analysis. Differences in cumulative dendritic spine distribution were evaluated using the Kolmogorov–Smirnov (K-S) test as described earlier (Dumitriu et al., 2012). For behavioral studies, statistical tests were conducted in SigmaPlot 12.5 Software. Student’s *t* test was used to compare two independent datasets. For multiple comparisons, we used ANOVA, followed by Dunnett’s test. Differences between genotypes or samples were considered significant at *p* < 0.05. In figures and table, data are reported as mean ± SEM, unless otherwise stated.

## Results

### Lack of CentA1 does not influence gross brain morphology

We generated CentA1 KO mice by replacing exon 3 (functional domain) of the *Centa1* locus with sequences for LacZ, followed by a translational STOP, and a neo selection cassette flanked by *frt* sites. Removal of the neo selection cassette was achieved by crossing with the *Flp* line. This modified locus leads to a fusion protein consisting of the beginning of CentA1 (exons 1 and 2) and LacZ, while the functional protein is missing (Fig. 1*A*). To assess whether CentA1 deletion affects gross brain structure, we compared the overall brain morphology between KO mice (CentA1 KO) and their NTG littermates. NeuN immunostaining of coronal sections was indistinguishable between genotypes (Fig. 1*B*). Lack of CentA1 expression in the brain (hippocampus, cerebellum, and cortex) of CentA1 KO mice was validated by immunoblotting (Fig. 1*C*). The expression level of Centγ3, a closely related Centaurin isoform highly enriched in the brain, showed a slight but not significant increase (26%; *p* = 0.29) in the hippocampus of CentA1 KO mice (Fig. 1*D*). Because CentA1 has been identified as an Arf6 GAP (Venkateswarlu et al., 2004), we evaluated the level of active (GTP bound) Arf6 in the hippocampal tissue of the CentA1 KO mice and their NTG littermates. We found a 300% increase in active Arf6 (as measured by active Arf6 pulled down with the GGA3-PBD) in the hippocampus of CentA1 KO mice compared with NTG littermates (Fig. 1*E*). This finding is consistent with CentA1 function as an Arf6 GAP protein.

### Loss of CentA1 leads to progressive increase in dendritic spine density in the hippocampus

**Figure 2.**
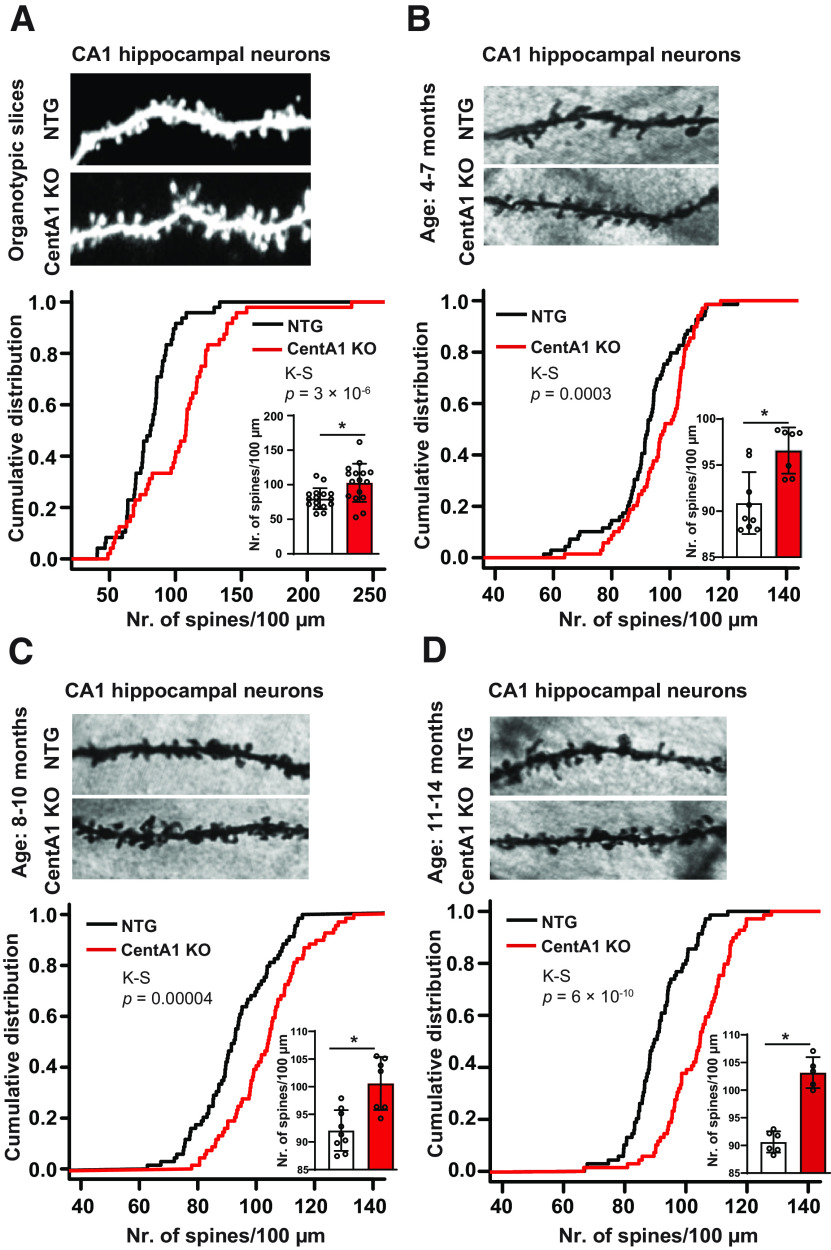
Lack of CentA1 protein leads to increased dendritic spine density in the hippocampus. ***A***, Representative images of apical dendrites from CA1 pyramidal neurons in organotypic slices from CentA1 KO and NTG mice. Slices were prepared from postnatal day 6 mice and cultured for one to two weeks. Neurons were gene gun transfected with eGFP and two-photon laser scanning microscopy imaged at DIV12. Cumulative frequencies were plotted using each analyzed dendrite from all animals in a genotype group. The inset bar graph shows the average spine density calculated per animal and per genotype group (*p = 0.006; *t*-test). NTG: *n* = 16 neurons of seven mice, KO: *n* = 16 neurons of 11 mice. Error bars indicate SEM. The average apical dendritic spine density is significantly increased in the CentA1 KO slices. K-S test, *p* = 3e-6. ***B***, Representative images of apical dendrites in Golgi-stained CA1 pyramidal neurons from four- to seven-month-old CentA1 KO mice and their NTG littermates. Cumulative frequencies were plotted using each analyzed dendrite from all animals in a genotype group. The inset bar graph shows the average spine density calculated per animal and per genotype group (*p = 0.002; *t*-test). NTG: *n* = 45 neurons of nine mice, KO: *n* = 35 neurons of seven mice. Error bars indicate SEM. Average apical dendritic spine density is significantly increased in the neurons of CentA1 KO mice. K-S test, *p* = 0.0003. ***C***, Representative images of apical dendrites in Golgi-stained CA1 pyramidal neurons from eight- to 10-month-old CentA1 KO mice and their NTG littermates. Cumulative frequencies were plotted using each analyzed dendrite from all animals in a genotype group. The inset bar graph shows the average spine density calculated per animal and per genotype group (*p = 0.001; *t*-test). NTG: *n* = 45 neurons of nine mice, KO: *n* = 35 neurons of seven mice. Error bars indicate SEM. Average apical dendritic spine density is significantly increased in the neurons of CentA1 KO mice. K-S test, *p* = 0.00004. ***D***, Representative images of apical dendrites in Golgi-stained CA1 pyramidal neurons from 11- to 14-month-old CentA1 KO mice and NTG littermates. Cumulative frequencies were plotted using each analyzed dendrite from all animals in a genotype group. The inset bar graph shows the average spine density calculated per animal and per genotype group (*p = 0.001; *t*-test). NTG: *n* = 30 neurons of six mice, KO: *n* = 25 neurons of five mice. Error bars indicate SEM. Average apical dendritic spine density is significantly increased in the neurons of CentA1 KO mice. K-S test, *p* = 6 × 10^−10^.

CentA1 is involved in small GTPase signaling, which is important for the maintenance and plasticity of dendritic spines (Woolfrey and Srivastava, 2016; Mignogna and D'Adamo, 2018). Previously we reported that overexpression of CentA1 decreases spine density, and shRNA-mediated downregulation of CentA1 rescues dendritic spines from Aβ-mediated elimination in CA1 pyramidal neurons (Szatmari et al., 2013). Dendritic spine density was evaluated on secondary apical dendrites from hippocampal CA1 neurons in NTG and CentA1 KO organotypic slices transfected with eGFP (prepared at postnatal days 5–7 and cultured for 6–7 d; Fig. 2*A*). We analyzed 16 pyramidal neurons per genotype (*n* = 7–11 animals/group). In CentA1 KO hippocampal slices, spine density was enhanced by 22.30% compared with NTG littermates (number of spines/100 μm; CentA1 KO = 102.83 ± 6.9; NTG = 79.8 ± 3.8; *p* = 3 × 10^−6^, K-S test). To examine whether the increase in spine density observed in organotypic slices from young animals also occurs *in vivo* in older mice, we evaluated spine density in hippocampal CA1 neurons of adult mice (aged 4–14 months), on Golgi-stained brain sections from CentA1 KO mice and their NTG littermates. Five neurons/animal were included in analysis (*n* = 5–8 animals/genotype/age group). Spine density in mice lacking CentA1 was significantly higher across all age groups (number of spines/100 μm; four to seven months: CentA1 KO = 96.6 ± 0.95; NTG = 90.9 ± 1.1; *p* = 0.0003; eight to 10 months: CentA1 KO = 103.5 ± 1.1; NTG = 92 ± 1.2; *p* = 0.00004; 11–14 months: CentA1 KO = 103.2 ± 1.3; NTG = 90.6 ± 0.8; *p* = 6 × 10^−10^, K-S test; Fig. 2*B–D*).

### Loss of CentA1 leads to enhanced spine plasticity and LTP

**Figure 3.**
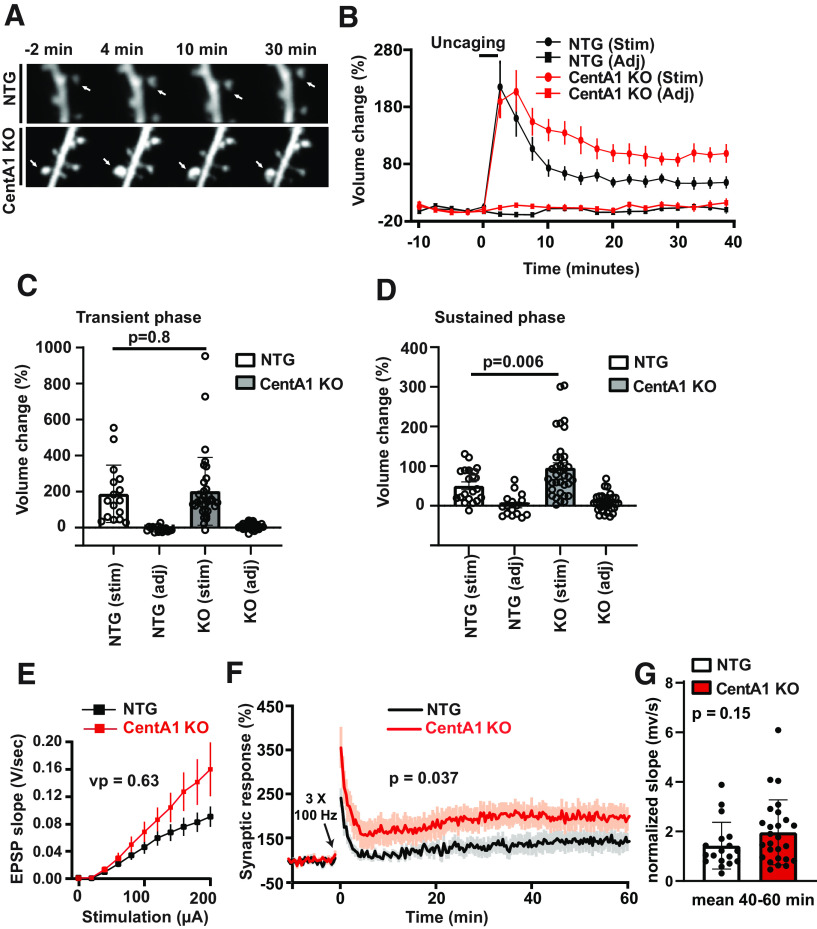
Lack of CentA1 enhances dendritic spine structural plasticity in the hippocampus. ***A***, Time-lapsed images of spine structural plasticity induced by two-photon glutamate uncaging in CentA1 KO and NTG neurons transfected with eGFP and imaged 4–6 d later. The arrows indicate stimulated spines. Structural plasticity was induced by applying a low-frequency train of two-photon uncaging pulses (6 ms, 30 pulses, 0.5 Hz) to a single dendritic spine in zero extracellular Mg^2+^ and 2 mm MNI-caged glutamate. ***B***, Time course of spine volume change in stimulated spines or adjacent spines (Adj) in CentA1 KO and NTG neurons. The number of samples (spine/neuron/mice) was 19/13/7 for NTG slices and 36/17/11 for KO slices. ***C***, Transient spine volume change (volume change averaged over 25–30 min subtracted by volume change at 2 min; p = 0.8. Error bars indicate SEM. ***D***, Sustained spine volume change (volume change averaged over 25–30 min.). Error bars indicate SEM. Two-tailed unpaired *t* test, *p* = 0.006. ***E***, Electrophysiological recordings showing average input/output curve of CentA1 KO [*n* (mice/experiments) = 12/26] and NTG (*n* = 9/19) littermate mice. Error bars indicate SEM. Non-parametric RM-ANOVA, *p* = 0.63. ***F***, Time course of change in synaptic response before and after LTP induction in CA1 pyramidal neurons from CentA1 KO [*n* (animals/experiments) = 12/26] and NTG (*n* = 9/19) littermate mice. Error bars indicate SEM. Non-parametric RM-ANOVA, *p* = 0.037. ***G***, Quantification of average potentiation (40–60 min) of neurons in F. Mean and SEM are shown. Mann–Whitney’s *U* test, p = 0.1501.

As dendritic spine number is an indicator of excitatory synapse density and of plasticity of individual synapses, we reasoned that lack of CentA1 might affect structural plasticity of individual dendritic spines. Therefore, we performed two-photon glutamate uncaging mediated single synapse stimulation on CA1 pyramidal neurons from CentA1 KO and NTG littermate organotypic hippocampal slices transfected with EGFP (Fig. 3). In NTG neurons, spine volume rapidly increased (∼1 min; reaching peak at 2–4 min) following glutamate uncaging (transient phase of spine plasticity; Fig. 3*A-C*) by 187 ± 39.8% and relaxed to an elevated level at 50.3 ± 9.7%, lasting >30 min (sustained phase of spine plasticity; Fig. 3*B*,*D*). In CentA1 KO neurons, glutamate uncaging induced a 202 ± 32.3% increase in spine volume during the transient phase that was not statistically significant compared with the NTG neurons (*p* = 0.8; Fig. 3*A–C*). However, the sustained phase of spine enlargement in CentA1 KO neurons was significantly higher than in NTG neurons (96 ± 12.6%; *p* = 0.006; Fig. 3*B*,*D*).

Since spine structural plasticity is considered to be a structural correlate of LTP (Matsuzaki et al., 2004) we also assessed the effect of CentA1 KO on LTP of fEPSP in Schaffer collateral–CA1 synapses in the hippocampus. LTP was elicited by applying a high-frequency stimulation protocol consisting of three trains of 100 Hz, separated by intervals of 20 s. As shown on Figure 3*E–G*, the LTP curve collectively analyzed is significantly different between genotypes, with CentA1 KO mice showing enhanced LTP [nonparametric repeated-measure (RM)-ANOVA, *p* = 0.037]. CentA1 KO mice also showed a trend toward enhanced LTP at 40- to 60-min time point, although this change was not statistically significant (*p* = 0.15; Mann–Whitney’s *U* test).

### CentA1 KO mice show normal synaptic ultrastructure

**Figure 4.**
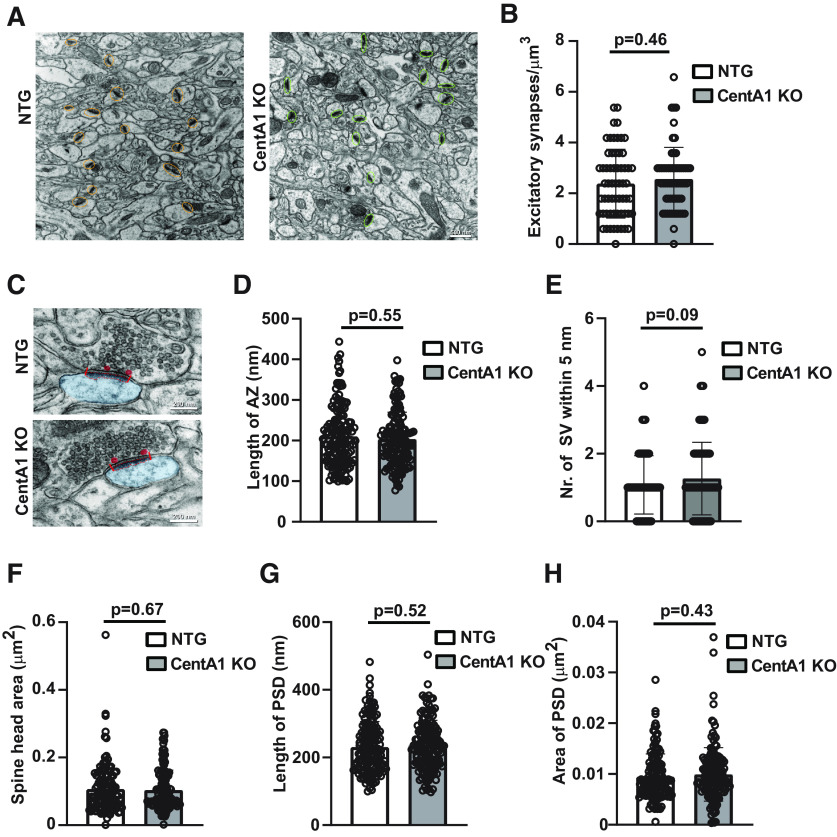
CentA1 KO mice exhibit normal synaptic ultrastructure in hippocampal CA1 neurons. ***A***, Representative EM images showing spine synapses in the middle of the SR of CA1 hippocampus from NTG (left) and CentA1 KO mice (right). Scale bar = 500 nm. ***B***, Graph compares spine synapse density in hippocampal sections from CentA1 KO mice and NTG at seven to eight months of age using the physical dissector method (three mice/genotype; *n* = 239 axospinous synapses from 20 micrographs for NTG and *n* = 257 axospinous synapses from 20 micrographs for CentA1 KO). ***C***, Representative EM images of spine synapses in the middle of the SR of the CA1 hippocampus from NTG (top) and CentA1 KO mice (bottom). Scale bar = 200 nm. ***D, E***, Quantification of mean AZ length and the number of docked SVs (within 5 nm of the presynaptic membrane) in CA1 hippocampus of CentA1 KO mice and NTG at seven to eight months of age (three mice/genotype; *n* = 50 synapses/animal). ***F***, Graph compares the size of dendritic spine head in hippocampal sections from CentA1 KO mice and NTG at seven to eight months of age (three mice/genotype; *n* = 50 synapses/animal). ***G, H***, Quantification of mean PSD length (between red lines) and area (demarcated with dotted red line) in CA1 hippocampus of CentA1 KO mice and NTG at seven to eight months of age (three mice/genotype; *n* = 50 synapses/animal). All data are presented as mean ± SEM. Unpaired *t* test showed no significant difference between genotypes.

To evaluate whether lack of CentA1 affects synapse ultrastructure, we analyzed spine synapse density in the SR of the hippocampus CA1 region and the fine structure of synapses using EM. We found no significant difference between genotypes in spine synapse density, presumably because of the limited number of samples we could measure for EM (NTG: 2.4 ± 0.17 μm^3^ vs CentA1 KO: 2.6 ± 0.16 μm^3^, *p* = 0.4608; Fig. 4*A*,*B*). Spine synapse ultrastructure was not changed in CentA1 KO mice when compared with NTG littermates (Fig. 4*C–H*). Specifically, we analyzed presynaptic ultrastructure by measuring the lengths of AZ (NTG: 210 ± 6.056 nm vs CentA1 KO: 203 ± 5.43 nm; *p* = 0.55; Fig. 4*D*) and the number of docked synaptic vesicles (NTG: 1.1 ± 0.07 vs CentA1 KO: 1.3 ± 0.09; *p* = 0.1; Fig. 4*E*). Postsynaptic ultrastructure was also analyzed by measuring spine head size (NTG: 0.107 ± 0.006 μm^2^ vs CentA1 KO: 0.104 ± 0.005 μm^2^; *p* = 0.67; Fig. 4*F*), length of PSD (NTG: 231 ± 6.17 nm vs CentA1 KO: 236.3 ± 5.98; *p* = 0.52; Fig. 4*G*) and area of PSD (NTG: 0.095 ± 0.0004 μm^2^ vs CentA1 KO: 0.1 ± 0.0004 μm^2^; *p* = 0.43; Fig. 4*H*). In all these parameters, we found no difference between genotypes.

### CentA1 KO mice exhibit improvement in hippocampus-dependent memory

An increase in spine density and plasticity in CentA1 KO may cause behavioral phenotypes. Thus, we examined the behavior of CentA1 KO mice on several standard tasks. Baseline behaviors in CentA1 KO mice and their NTG littermates were evaluated for control purposes and showed no significant difference between genotypes (Table 1). CentA1 KO mice and NTG littermates show similar levels of activity in the OF and similar performance in EPM test, which are viewed as indicators of anxiety-like behavior. Performance on the accelerating RR was similar, indicating normal coordination and balance in the absence of CentA1. Working memory, as measured by spontaneous alteration test, was also intact in CentA1 KO mice. Therefore, mice lacking CentA1 have normal levels of motor activity, anxiety-like behavior, and working memory.

**Table 1:** Baseline behaviors in CentA1 null (*n =* 23) and CentA1 NTG littermates (*n* = 28)

Behavioral test	Measurement	Testing for	CentA1 KO	NTG	Statistical significance	Statistical test
OF	Distance moved (cm)	Activity levels	2964.3 ± 140.8	2794.57 ± 137.67	*p* = 0.39 (not significant)	*t* test
OF	Exit latency (s)	Anxiety	3.72 ± 0.70	2.45 ± 0.53	*p* = 0.15 (not significant)	*t* test
EPM	Time spent in open arm (%)	Anxiety-like behavior	16.56 ± 4.10	22.90 ± 3.82	*p* = 0.26 (not significant)	*t* test
RR	Time spent on rod over three trials (s)	Motor coordination and balance	123.67 ± 10.26	139.58 ± 9.16	*p* = 0.25 (not significant)	*t* test
MWM	Swim speed (cm/s)	Motor ability	18.81 ± 0.62 cm/s (probe trial)	20.09 ± 0.47 cm/s (probe trial)	*p* = 0.11 (not significant)	*t* test
MWM-VPT	Latency to visible platform (s)	Visual acuity	18.28 ± 1.35	18.15 ± 1.28	*p* = 0.94 (not significant)	*t* test
SA	Alternation success between two mazes (%)	Working memory	66.67 ± 10.54	80.00 ± 8.16	*p* = 0.33 (not significant)	*t* test

Table shows the behavioral tests that were performed to identify potential abnormalities in baseline behaviors of CentA1 KO mice. On all measures, there was no statistically significant difference between the CentA1 KO mice and their wild-type littermates.

**Figure 5.**
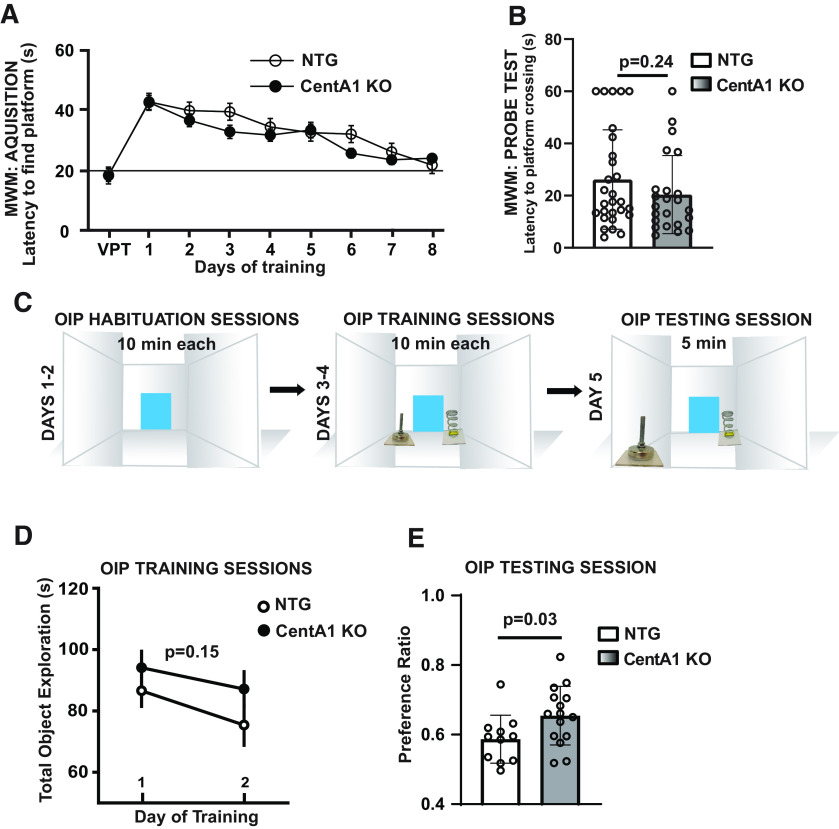
Behavioral evaluation of CentA1 KO mice. ***A–B***, Morris Water Maze test shows no significant difference between genotypes in latency to platform during acquisition phase (***A***) or during probe test (***B***) (CentA1 KO; *n* = 28 and NTG littermates; *n* = 23). ***C***, Diagram of OIP memory test, ***D***, Graph shows no significant difference between genotypes in the average duration of object exploration during training (CentA1 KO; *n* = 15 and NTG littermates; *n* = 11). ***E***, CentA1 KO mice exhibit significant discrimination of the familiar and novel object locations and exhibit a strong preference for the object in the novel location as compared with their NTG littermates (*p* = 0.03) during OIP testing session. All data are presented as mean ± SEM.

As the signaling pathways regulated by CentA1 (including ERK, Ras, and Arf6) have been shown to mediate certain hippocampus-dependent memory (Ye and Carew, 2010; Oku and Huganir, 2013; Lee, 2014; Kim et al., 2015; Tagliatti et al., 2016), we asked whether CentA1 might be involved in these functions. We started with the MWM task, a commonly used test for hippocampus-dependent learning and memory. We evaluated swim speed and latency to the platform in the visible platform version of the MWM and found no difference between genotypes (Table 1). Next, we trained CentA1 null and NTG littermates using four trials/day for 8 d, with an intertrial interval of 20–30 min. As expected, the latency to find the platform decreased with training in both genotypes (Fig. 5*A*). On day 9, we administered a probe test. Although CentA1 KO mice displayed shorter latency to platform, the difference between genotypes was not statistically significant (as mean latency: CentA1 KO = 20.4 ± 3.27, NTG = 26.17 ± 3.81; *p* = 0.24; Fig. 5*B*).

To assess phenotypes corresponding to increased spine density and plasticity of CentA1 KO, we set out to perform a test with higher demand on hippocampus-dependent memory. We chose the OIP memory task, since it is a well-characterized test of hippocampal function and requires the mice to remember multiple different objects in specific locations, placing a high load on spatial memory. The OIP test depends on the interaction between the hippocampus, perirhinal cortex, and medial prefrontal cortex (Barker and Warburton, 2015). Briefly, we trained mice to recognize the location change for one of two familiar objects, located in a familiar arena. If the mouse has recognized the location change, it will show a higher exploration preference for the object moved to a new location. The experimental design of OIP test is presented in Figure 5*C*. CentA1 KO mice and their NTG littermates were habituated to the arena for 10 min/d for two consecutive days. This was followed by 2 d of training that consisted of 10 min/d in the arena that now contained two toy objects. During the OIP training session, there was no difference in total object exploration time (s) between genotypes (mean total object exploration for the first training session: CentA1 KO = 94.09 ± 5.38, NTG = 86.58 ± 5.11; *p* = 0.34; mean total object exploration for second training session: CentA1 KO = 87.13 ± 5.68, NTG = 75.37 ± 6.60; *p* = 0.15; Fig. 5*D*); 24 h later, we performed a 5-min OIP testing session in the familiar arena, with one toy object transferred to the opposing southern corner (Fig. 5*E*). We found that CentA1 KO mice preferred the object in a novel location significantly more than the NTG littermates (preference ratio: CentA1 KO = 0.65 ± 0.02, NTG = 0.59 ± 0.02; *p* = 0.03; Fig. 5*E*), which indicates that associative recognition memory is significantly enhanced in mice lacking CentA1.

Taken together, our findings suggest that in the normal brain, CentA1 negatively regulates dendritic spine density and structural plasticity. Moreover, inhibition of CentA1 signaling enhances hippocampus-dependent spatial memory formation.

## Discussion

In the present study, we report that ADAP1/CentA1 functions as a negative regulator of dendritic spine density, spine structural plasticity in the hippocampus, and hippocampus-dependent spatial memory formation. *Centa1* null mice show age-dependent enhancement in dendritic spine density (Fig. 2) in the hippocampal CA1 neurons and improved performance in the OIP spatial memory task (Fig. 5*C–E*). These mice also displayed enhanced Arf6 activity (Fig. 1), which, together with an increased number and enhanced structural plasticity of dendritic spines, might represent a possible cellular mechanism for the observed behavioral phenotype. Our finding that deletion of CentA1 enhances spine density indicates a central role for CentA1 signaling in spinogenesis, spine elimination, or maturation. Interestingly, transient reduction of the CentA1 level using an shRNA approach did not significantly increase spine density (Szatmari et al., 2013). This discrepancy can be explained by the developmentally regulated level of CentA1 in the brain, with the highest expression before neuronal maturity is achieved (Moore et al., 2007). Importantly, we also observed an enhanced spine structural plasticity in CentA1 KO. Since spine structural plasticity is related to the stabilization of newly formed spines during development (Zito and Svoboda, 2002), CentA1 deletion may have enhanced the stability of dendritic spines, leading to a higher density of dendritic spines. The enhanced spine density and spine structural plasticity in CentA1 KO may have been caused by its downstream GTPase molecules Ras (Hayashi et al., 2006; Szatmari et al., 2013) and Arf6 (Kim et al., 2015). The deletion of CentA1 leads to higher Arf6 activation (Fig. 1*E*) and lower Ras activation (Hayashi et al., 2006; Szatmari et al., 2013). Since Ras is required for inducing synaptic plasticity and stabilization (Zhu et al., 2002; Zhang et al., 2018), it may be difficult to explain our results by reduction of Ras. On the other hand, it has been reported that Arf6 can regulate spine density in either a positive or negative direction, depending on the developmental stage (Kim et al., 2015). Since early inhibition trends to increase spine density (Kim et al., 2015), Arf6 may mediate the gain of spine density in CentA1 KO. In addition to changes in these signaling pathways, it is known that CentA1 can regulate actin cytoskeleton via interaction with F-actin (Thacker et al., 2004) and microtubule-binding proteins, such as KIF13B (Kanamarlapudi, 2005; Venkateswarlu et al., 2005). Further analyses are necessary to identify downstream signals linking CentA1 and negative effects on spine density and plasticity.

Previous studies reported that changes in dendritic spine number and morphology correlate with spatial learning and memory (Moser et al., 1994; Leuner et al., 2003; Leuner and Shors, 2004; Mahmmoud et al., 2015). We therefore performed a battery of behavioral testing to evaluate whether enhanced spine density and structural plasticity result in improved learning and memory in mice lacking CentA1. The performance of CentA1 KO mice was comparable with NTG mice in the majority of the tests, including the MWM test (Fig. 5). Surprisingly, in the OIP associative recognition memory task, which requires the subject to associate an object with the place in which it was previously encountered, the CentA1 KO mice performed significantly better than control mice (Fig. 5). These results argue that CentA1, which is highly expressed in the hippocampus and prefrontal cortex, may play an important role in the neural circuits connecting the dorsal hippocampus, perirhinal cortex, and medial prefrontal cortex (Moser and Moser, 1998; Fanselow and Dong, 2010; Barker and Warburton, 2015).

## Synthesis

Reviewing Editor: Alfonso Araque, University of Minnesota Decisions are customarily a result of the Reviewing Editor and the peer reviewers coming together and discussing their recommendations until a consensus is reached. When revisions are invited, a fact-based synthesis statement explaining their decision and outlining what is needed to prepare a revision will be listed below. The following reviewer(s) agreed to reveal their identity: Anne Theibert, Venkateswarlu Kanamarlapudi. Note: If this manuscript was transferred from JNeurosci and a decision was made to accept the manuscript without peer review, a brief statement to this effect will instead be what is listed below.

The reviewers have found merit and interest on the findings reported. I concur with the reviewers’ comments expressing the interest of the study. However, they also expressed some concerns about several issues that need to be addressed and clarified. I also consider pertinent the concerns expressed.

Reviewers and I believe that addressing the comments requires less than two months of work. Please, let me know if you need extra time.

I am looking forward to receiving a revised version of the manuscript with the reviewers´ comments addressed.

Comments of the reviewers:

Reviewer 1.

Line 360 The authors state “that lack of CentA1 does not lead to compensatory upregulation of Centγ3” but without any quantification of data or data not shown.

Line 364-366 The authors state, “We found a 300% increase in basal Arf6 activity (as measured by active Arf6 pulled down with the GGA3 protein-binding domain (GGA3-PBD) in the hippocampus of CentA1 KO mice compared to NTG (FIG. 1E).” However, since the level of Arf6-GTP was assessed from in vivo tissue, it is not possible to know whether this was basal or stimulated Arf6 and the amount of stimulation. Thus it should be stated as “the level of active (GTP bound) Arf6 in hippocampal tissue.”

Beginning Line 368 Figures 2 and 3

• For the dendritic spine analysis: “Spine density” is more accurately presented in cumulative probability distributions of the “# of spines per dendritic segment", binned from the lowest density to the highest density segments. Then, those distributions can be compared with KS (Kolmogorov-Smirnov non-parametric test that compares 2 cumulative probability distributions) or a similar test. The reason being is that “spine density per segment” is a highly variable measure (not normally distributed) not well represented by the mathematical average. The more accurate type of analysis is shown in Dumitreu et al 2012 J. Neurosci Vol 32 (20) p 6957-6966.

• The rationale to examine CA1 neurons for spine analysis was not provided.

• The authors conclude that “Loss of CentA1 leads to progressive increase in dendritic spine density in the hippocampus.” The analysis of spine density was performed in cultured organotypic slices from P6-7 mice cultured for one week prior to biolistic transfection. They showed an enhancement of 22.3%. Analysis of Golgi stained sections from mice between 4-14 months showed an approximate two fold increase in spine density increase (from ∼6% to 12% increase). (These values should be provided in the text). Since it’s not accurate to directly compare the results obtained using the two different techniques, and since it’s not feasible to culture organotypic slices from older mice, then the analysis of spine density should also be performed using the Golgi technique on early postnatal mice. (If one compared the results from the two types of experiments directly, it would suggest a bimodal effect with the knock out showing an initial large postnatal effect on spine density followed by a developmental decrease in the magnitude of the effect followed by a later increase.)

• For a newly characterized knock out mouse, it would be expected that dual immunolabeling for a presynaptic marker and a postsynaptic marker for excitatory synapses (VGLUT and PSD-95, or Homer), on top of the GFP fluorescence from the spine. This will allow an estimation of excitatory synapses on spines, vs. “empty” spines.

• The effects should not be described as being “larger” or “higher” or values being “enhanced” when there’s no statistical significance in those comparisons.

• The bar graphs need to show the individual data points, not just the mean {plus minus}SD (or SEM), or be replaced by scatter plots. There is something wrong with the bar graphs showing the difference in LTP (Fig. 3G).

• It is unclear how the areas of the PSD were determined. Is this derived from single sections, or is that value just the area enclosed by the perimeter of the PSD observed side-ways on a single EM section? Usually, “area of PSD” refers to the area of the whole disk as obtained from 3D reconstructions of serial EM sections.

• The authors need to describe the apparently different Input/Output relationships for field EPSP (Fig. 3E steeper curve in KOs?).

Line 417 and Figure 5

Four recognition memory tasks have been described in the literature: novel object preference, object location, object-in-place and temporal order. The rationale for choosing the object-in-place should be provided. This test should be referenced in the methods as well.

486 The authors state that “Arf6 may mediate the gain of spine density in CentA1 KO.” While it is a possibility that the effects of CentA1 KO on spines are mediated through Arf6 regulation, since CentA1 is an Arf6 GAP, it would be expected that loss of CenA1 would lead to increased active (GTP bound) Arf6 as shown in Figure 1. However, Kim et al. 2015 concluded that “ARF6 activation increased the spine formation in developing neurons, whereas it decreased spine density in mature neurons. Thus it seems possible that the loss of CentA1 affects spine density through one of its many other interactions. This point should be reconciled with the reference.

General Comments:

The authors need to justify the use of males only.

The following were not addressed: blinding, randomization, inclusion and exclusion criteria, and statistical sower given the chosen sample number.

The comparison control groups for knock out mice are usually referred to as “wild-type mice.” In this study the comparison mouse group are “non-transgenic controls (NTGs).” This should be explained.

Since there was no difference in the control and KO mice in the well-characterized Morris Water Maze, this test does not need to be described in the results.

Both LTP and LTD have been implicated in spatial recognition memory. Why were only LTP studies performed or shown?

What is the implication of the specificity of the OIP result compared with other spatial memory tests. Have other genetically modified or pharmacologically treated mice shown no effects on other spatial memory tasks but specifically on the OIP task?

Reviewer 2.

ADAP1/Centaurin-α1 (CentA1) is an Arf6 GAP, which is known as a regulator dendritic differentiation and a potential mediator of Alzheimer’s disease (AD) pathogenesis. In this manuscript, the authors analysed the functions of Centaurin-α1 in the brain by using the CentA1 knockout mouse. They found that Centaurin-α1 functions as a negative regulator of spine density and plasticity, and hippocampus-dependent memory formation. It is a well-written manuscript with appropriate methods used and clear data. However, addressing the following comments strengthen the manuscript.

1. Fig. 1: Fig. 1C, please specify what 5 lanes represent in the control NTG group (5 mice?) in the figure legend (C, Immunoblots show the complete lack of CentA1 protein in the hippocampus from CentA1 KO mice (NTG = 5 mice and KO = 3 mice)). Also, include a graph showing quantification of Arf6 activation from (E). Also, correct ‘IP’ to ‘PD’ in the top blot in Fig. 1E and also specify what ‘PD’ (pull-down) stands for in the figure legend.

2. Fig. 2: Please show representative images of apical dendrites in Golgi stained CA1 pyramidal neurons from 4-7 months and 11-14 months old CentA1 KO mice and NTG littermates.

3. Fig. 3: Kindly increase the size of the symbols in Fig. 3B and Fig. 3E so that it easy to distinguish NTG and CentA1 KO groups.

4. There are few typos and grammatical mistakes in the manuscript that require attention.

## Author Response

Response to Reviewer 1

We thank to reviewer for the supportive comments and recommendations to further improve the revised manuscript.

1.) In response to a previous comment, the authors reply: “We changed the wording in the text of the manuscript as follows:”... the level of active (GTP bound) Arf6 in hippocampal tissue” (Line 363-364).”

However in the subsequent lines (365-367) the authors state: “We found a 300% increase in BASAL Arf6 activity (as measured by active Arf6 pulled down with the GGA3 protein binding domain (GGA3-PBD)in the hippocampus of CentA1 KO mice compared to NTG littermates (FIG. 1E).” This should be revised to ACTIVE Arf6.

As suggested, we changed the wording in the text of the manuscript as follows:

"We found a 300% increase in active Arf6 activity (as measured by active Arf6 pulled down with the GGA3 protein-binding domain (GGA3-PBD) in the hippocampus of CentA1 KO mice compared to NTG littermates (FIG. 1E). (Line 364-367)

2.) Lines 378-379 “...compared with NTG littermates (number of spines/100 μm; CentA1 KO = 102.83{plus minus}6.9; NTG =79.8{plus minus}3.8; p = 3e-6).”

And Lines 385-386: “... 10 months: CentA1 KO = 103.5 {plus minus} 1.1; NTG = 92{plus minus}1.2; p = 0.00004; 11-14 months: CentA1 KO = 103.2

386{plus minus}1.3; NTG = 90.6{plus minus}0.8; p = 6E-10).”

Figure 1D p = 6e-10

It’s not clear what these p values are specifying; the p values need to be revised.

In order to clarify these points, we added “K-S test” (Kolmogorov-Smirnov test, defined in line 344) after the p values. In addition, we changed the expression of exponent from E-notation (6e-10) to sciuentific notation (6 × 10-6) in the main text and Figure 1D.

"(number of spines/100 μm; CentA1 KO = 102.83 {plus minus}6.9; NTG = 79.8{plus minus}3.8; p = 6 × 10-6, K-S test).” (Line 377-378)

"(FIG. 2B-D; number of spines/100 μm; 4-7 months: CentA1 KO = 96.6 {plus minus}0.95; NTG = 90.9{plus minus}1.1; p = 0.0003; 8-10 months: CentA1 KO = 103.5 {plus minus}1.1; NTG = 92{plus minus}1.2; p = 0.00004; 11-14 months: CentA1 KO = 103.2 {plus minus}1.3; NTG = 90.6{plus minus}0.8; p = 6 × 10-10, K-S test)” (Line 384-385)

3.) The authors respond to “Since there was no difference in the control and KO mice in the well-characterized Morris Water Maze, this test does not need to be described in the results.”

We disagree with this. Although there is no behavioral difference between the tested genotypes in terms of training or probe of the MWM, the data should not be discounted. It may not need to be emphasized in the results but just because there is no difference, does not mean the data is not informative...”

The comment in the first review meant that the TEST does not need to be described, and was not referring to the RESULTS/DATA, which should be provided. Since the MWM is a well-characterized test, it is not necessary to describe the test in the results. Moreover, the other behavioral tests are not described in the results section, only the results/data. The recommendation is to remove, starting with Line 439 “Briefly, the mouse is trained to find a submerged platform based on visual cues placed on the walls of the training room. After training, the platform is removed, and a probe test is conducted to evaluate spatial learning. If the mouse has learned a spatial search strategy, it will spend more time in the target quadrant and will display more crossings at the place where the escape platform was located.” The results/data could begin with “We evaluated swim speed and latency...” This editing would provide valuable word space to further highlight/explain the differences between the MWM (and other tests) and the OIP test, that support the possibility that “[CenA1] may play an important role in the neural circuits connecting the dorsal hippocampus, perirhinal cortex and medial prefrontal cortex.”

We followed the recommendation of the reviewer and shortened the description of MWM test and the results as follows:

"We started with the Morris water maze (MWM) task, a commonly used test for hippocampus-dependent learning and memory. We evaluated swim speed and latency to the platform in the visible platform version of the MWM and found no difference between genotypes (Table 1). Next, we trained CentA1 null and NTG littermates using four trials/day for 8 days, with an intertrial interval of 20-30 minutes. As expected, the latency to find the platform decreased with training in both genotypes (FIG. 5A). One day 9, we administered a probe test. Although CentA1 KO mice displayed shorter latency to platform, the difference between genotypes was not statistically significant (FIG. 5B, as mean latency: CentA1 KO = 20.4{plus minus}3.27, NTG = 26.17{plus minus}3.81; p = 0.24).” (Line 438-445)

As for the discussion of the brain region, in the previous manuscript, we clearly described in the discussion section as:

"These results argue that CentA1, which is highly expressed in the hippocampus and prefrontal cortex, may play an important role in the neural circuits connecting the dorsal hippocampus, perirhinal cortex and medial prefrontal cortex (Moser and Moser, 1998; Fanselow and Dong, 2010; Barker and Warburton, 2015).” (Line 510-513)

Since this is speculation, we feel it should be in the discussion, not in the result section.

4.) Line 496 should be “On the other hand,"

We made the correction as follows:

"On the other hand, it has been reported...” (Line 4
